# Redetermination of the structure of 2-amino-8-thia-1,5-di­aza­spiro­[4.5]dec-1-en-5-ium chloride monohydrate

**DOI:** 10.1107/S2056989022000111

**Published:** 2022-01-11

**Authors:** Lyudmila A. Kayukova, Elmira M. Yergaliyeva, Anna V. Vologzhanina

**Affiliations:** a JSC A. B. Bekturov Institute of Chemical Sciences, 106 Shokan Ualikhanov str., 050010, Almaty, Kazakhstan; b X-Ray Structural Centre, A.N. Nesmeyanov Institute of Organoelement Compounds, RAS, 28 Vavilova str., 119991 Moscow, Russian Federation

**Keywords:** crystal structure, hydrogen bonding, redetermination, spiro­[4.5]dec-1-en-5-ium, tosyl­ation

## Abstract

The reaction of β-(thio­morpholin-1-yl)propio­amidoxime with tosyl chloride in CHCl_3_ in the presence of DIPEA when heated at 343 K for 8 h afforded the title hydrated salt, C_7_H_14_N_3_S^+^·Cl^−^·H_2_O, in 84% yield. This course of the tosyl­ation reaction differs from the result of tosyl­ation obtained for this substrate at room temperature, when only 2-amino-8-thia-1,5-di­aza­spiro­[4.5]dec-1-ene-5-ammonium tosyl­ate was isolated in 56% yield. The structure of the reaction product was established by physicochemical methods, spectroscopy, and X-ray diffraction. The single-crystal data demonstrated that the previously reported crystal structure of this compound [Kayukova *et al.* (2021). *Chem. J. Kaz*, **74**, 21–31] had been refined in a wrong space group. In the extended structure, the chloride anions, water mol­ecules and amine groups of the cations form two-periodic hydrogen-bonded networks with the **fes** topology.

## Abstract 2

The crystal structure of the title compound was redetermined at two temperatures [120.0 (2) and 295.0 (2) K]. The previously reported *P*2_1_2_1_2_1_ chiral space group is corrected to centrosymmetric *Pbca*.

## Chemical context

Sulfochlorination of amidoximes is known to afford stable products of acyl­ation at the oxygen atom of the amidoxime group; at the same time, the sulfochlorination reaction of derivatives of primary amidoximes can, depending on the structure of the starting amidoxime and reaction conditions, lead to rearranged products with the formation of ureas and substituted cyanamides (Tiemann, 1891 ▸; Bakunov *et al.*, 2000 ▸; Doulou *et al.*, 2014 ▸) .

Previously, in our studies of the acyl­ation of β-amino­propio­amidoximes with acid chlorides of substituted benzoic acids, only *O*-acyl-β-amino­propio­amidoximes were identified as acyl­ation reaction products. Their structures have been determined by the complex use of spectroscopic methods, as well as X-ray structural analysis (Kayukova, 2003 ▸; Beketov *et al.*, 2004 ▸; Kayukova *et al.*, 2010*a* ▸). The dehydration of the products of the *O*-acyl­ation of β-amino­propio­amidoximes allows for 3,5-disubstituted 1,2,4-oxa­diazo­les to be obtained, which under conditions of acid hydrolysis and in the presence of moisture are capable of undergoing a Boulton–Katritsky rearrangement to 2-amino-1,5-di­aza­spiro­[4.5]dec-1-en-5-ium salts (Kayukova *et al.*, 2010*b* ▸, 2018 ▸, 2021*a* ▸).

**Figure 1:**
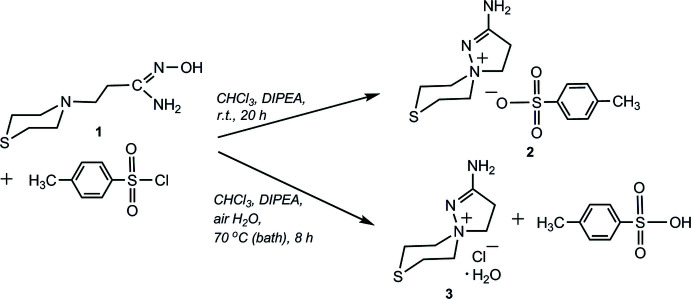
Results of the β-(thio­morpholin-1-yl)propio­amidoxime (**1**) tosyl­ation reaction at r.t. and at the boiling point of the solvent.

Recently, we found that the aryl­sulfochlorination reaction of β-amino­propio­amidoximes at room temperature afforded 2-amino-8-(hetera)-1,5-di­aza­spiro­[4.5]dec-1-en-5-ium aryl­sulfonates as the main products (Kayukova *et al.*, 2020 ▸, 2021*b* ▸). Herein we report on the result of β-(thio­morpholin-1-yl)propio­amidoxime tosyl­ation at the boiling point of the solvent. By means of such a high-temperature process, the formation of the most stable reaction product is expected. Under such conditions of thermodynamic control of the tosyl­ation reaction of β-(thio­morpholin-1-yl)propio­amidoxime (**1**) upon prolonged heating for 8 h at the boiling point of the solvent [CHCl_3_, 8 h, 343 K (bath temperature)], in the presence of DIPEA, the title hydrated salt, 2-amino-8-thia-1,5-di­aza­spiro­[4.5]dec-1-en-5-ium chloride monohydrate (**3**) was obtained in good yield (84%). In our opinion, the source of hydrate formation was air moisture, since the formation of single crystals took place over a long time under conditions of natural evaporation of the solvent for crystallization with air access. This result of the amidoxime (**1**) tosyl­ation differs from the result of the same reaction performed at room temperature, when the main kinetic product of the reaction was 2-amino-8-thia-1,5-di­aza­spiro­[4.5]dec-1-en-5-ium tosyl­ate (**2**) (Fig. 1 ▸, yield 56%; Kayukova *et al.*, 2021*b* ▸).

Spiro­pyrazolinium chloride monohydrate **3** is a white opaque precipitate, poorly soluble in chloro­form. When the reaction was complete, it was filtered off from the reaction mixture and recrystallized from propanol-2 solution over three weeks in the form of transparent prisms with a melting point of 575 K. We previously isolated a compound with the same chemical composition and melting point during the acid hydrolysis of 5-aryl-3-(β-thio­morpholino­eth­yl)-1,2,4-oxa­diazo­les (Kayukova *et al.*, 2010*b* ▸). Not only the composition, but also the ortho­rhom­bic unit-cell parameters were similar for **3** and the previously reported structure; however, the space groups were different: *P*2_1_2_1_2_1_ at room temperature (Kayukova *et al.*, 2010*b* ▸) and *Pbca* at 120.0 (2) K for **3**, thus a single crystal of the reaction product was also determined at 295.0 (2) K and the resulting crystal structures were compared with the previously reported one. 
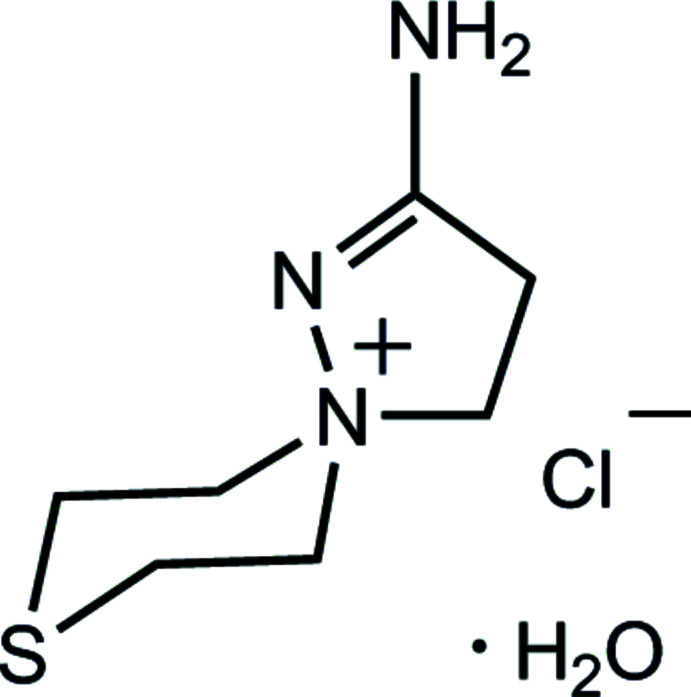


## Structural commentary

**Figure 2:**
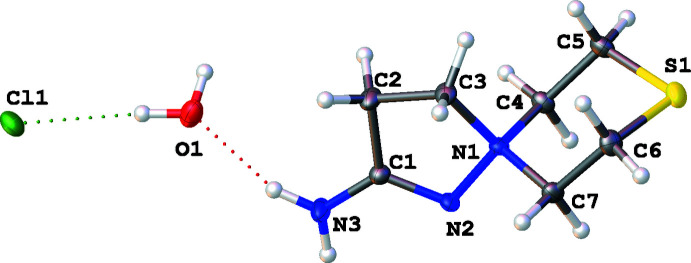
Asymmetric unit of **3** at low temperature with displacement ellipsoids at the 50% probability level.

The mol­ecular structure of **3** is shown in Fig. 2 ▸. The C3—N1 and N1—N2 bonds are elongated as compared with typical single bonds at 1.521 (1) and 1.463 (1) Å, respectively, which can be related to the anomeric effect of the lone pair of atom N2. The six- and five-membered rings of the C_7_H_14_N_3_S^+^ cation adopt chair and envelope conformations, respectively. It may be noted that in respect to a chair conformation of the six-membered ring, atom N2 can be situated in the equatorial and axial positions of the N1 atom; however, in this and previously reported salts, only the axial disposition of the N2 atom is observed. This is in accord with our B3LYP/6-31++G(d,p) calculations of standard Gibbs free energies of reactions leading to the formation of various products. We established that the axial stereoisomer is more stable than the equatorial one (Δ*G* = −144.29 and −124.23 kJ mol^−1^, respectively; Yergaliyeva *et al.*, 2021 ▸).

**Figure 3:**
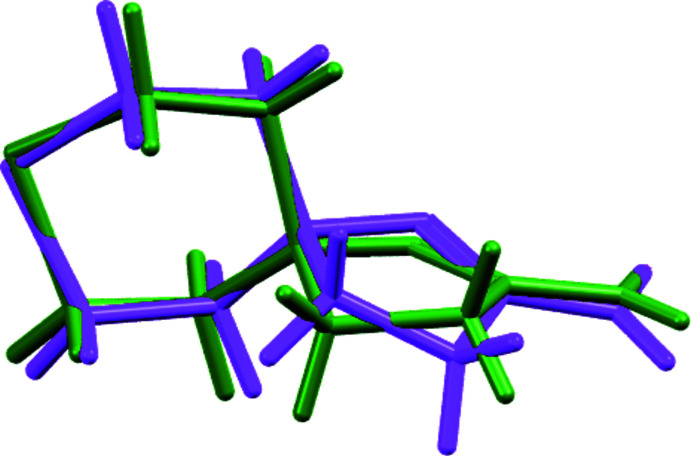
The two independent 2-amino-8-thia-1,5-di­aza­spiro­[4.5]dec-1-en-5-ium cations observed in APOBOX depicted as overlaid mol­ecules.

The envelope conformation of the C1/C2/C3/N1/N2 five-membered ring in **3** is expressed as the deviation of C3 from the mean plane formed by atoms N1/N2/C1/C2 (r.m.s. deviation = 0.005 Å): it is equal to 0.401 (1) Å, and the two mol­ecular conformers (corresponding to different directions of this carbon atom shifted in respect to the N—N=C—C mean plane) are equally present in this centrosymmetric crystal. However, the previously reported crystal structure (Kayukova *et al.*, 2010*b* ▸) [refcode APOBOX in the Cambridge Crystallographic Database (CSD; Groom *et al.*, 2016 ▸)] contains two independent spiro-cations in the asymmetric unit with two different conformations of the five-membered ring (Fig. 3 ▸). A question arises as to whether these structures are polymorphs of the same salt, or if the previously reported structure was incorrectly solved and refined. Our study of the same single crystal of **3** at room temperature confirmed that no phase transition occurs between 120 and 295 K. Unfortunately, crystallographic data for APOBOX stored in the CSD could not be re-refined. Thus, we compared the crystal packing and the system of hydrogen bonds for the two models refined in different space groups.

## Supra­molecular features

**Figure 4:**
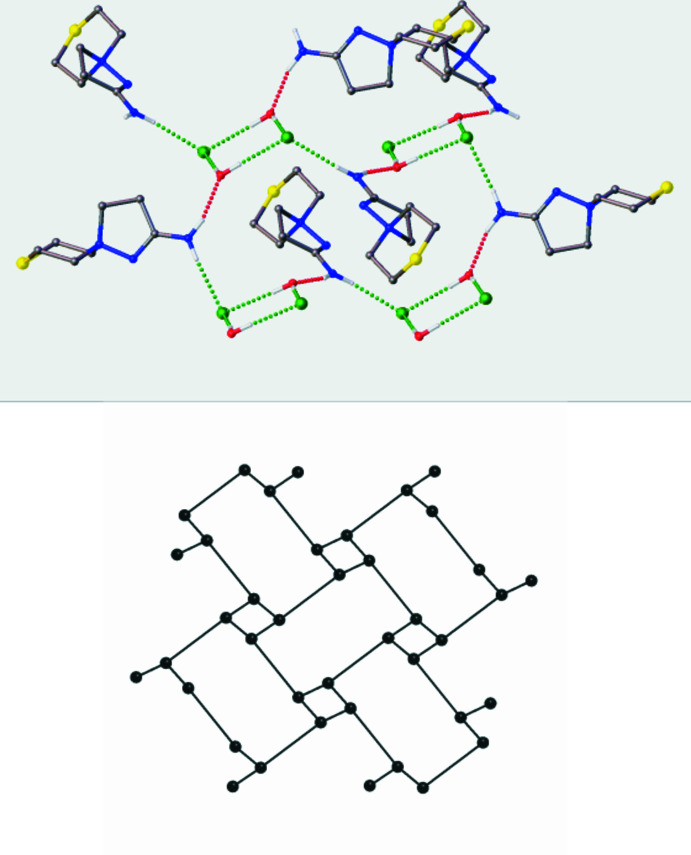
Top: fragment of the hydrogen-bonded layers in **3.** Hydrogen bonds are depicted as dotted lines. C-bound H atoms are omitted. Bottom: underlying net of hydrogen bonds in **3** with a **fes** topology.

First, the system of hydrogen bonds was compared for the two solids at 120.0 (2) and 295.0 (2) K (Tables 1 ▸ and 2 ▸) and they are essentially the same, apart from a slight lengthening of the H⋯*X* contacts at the higher temperature. In both cases, the amine acts as a donor of hydrogen bonds with a water mol­ecule, an anion and the water mol­ecules act as acceptors of N—H⋯O bonds and as donors in two O—H⋯Cl inter­actions, and the chloride anion is an acceptor of three hydrogen bonds. As a result, infinite layers parallel to the (001) plane are observed (Fig. 4 ▸). A topological analysis of the system of hydrogen bonds, where the spiro-cations act as linkers and water mol­ecules and anions are three-connected nodes, indicates that both layers are isoreticular and have the **fes** topology (the three-letter code is given in terms of the RSCR notation; O’Keeffe *et al.*, 2008 ▸).

Additional analysis of the crystal packing by means of the *PLATON* package (Spek, 2020 ▸) suggests that the *Pbca* space group is correct for both solids, and by means of the ‘Crystal Packing Similarity’ tool implemented within *Mercury* (Macrae *et al.*, 2020 ▸) as described by Childs *et al.* (2009 ▸) or by Vologzhanina (2019 ▸) denotes that the packings of 30-mol­ecule clusters for the two solids are also very close to each other (the average r.m.s. deviation of 0.15 Å can be explained by the different experimental temperatures). Thus, we propose that 2-amino-8-thia-1,5-di­aza­spiro­[4.5]dec-1-en-5-ium chloride monohydrate crystallizes in the *Pbca* space group both at low and room temperatures in contrast with the data given previously in space group *P*2_1_2_1_2_1_(Kayukova *et al.*, 2010*b* ▸).

## Synthesis and crystallization

IR spectra were obtained on a Thermo Scientific Nicolet 5700 FTIR instrument in KBr pellets. ^1^H and ^13^C NMR spectra were recorded on a Bruker Avance III 500 MHz NMR spectrometer (500 and 126 MHz, respectively). Melting points were determined on a TPL apparatus (Khimlabpribor, Russia). The progress of the reaction was monitored using Sorbfil TLC plates (Sorbpolymer, Russia) coated with CTX-1A silica gel, grain size 5–17 µm, UV-254 indicator. The spots were developed in I_2_ vapours and in the UV light of a chromatoscope (λ = 254 nm) TSX 254/365 (PETROLASER). The eluent for the analysis was a mixture of EtOH:benzene = 1:1 + a few drops of a 25% aqueous solution of NH_3_. Microanalysis according to the Pregl method was carried out on an elemental analyser with the absorption of CO_2_ and O_2_ isolated during combustion with a two-degree repetition of combustion.

The tosyl­ation of β-(thio­morpholin-1-yl)propio­amidoxime (**1**) was performed in dried CHCl_3_ with tosyl chloride in the presence of DIPEA, purchased from Sigma–Aldrich and used without purification. Solvents for synthesis, recrystallization and TLC analysis (EtOH, 2-PrOH, benzene, CHCl_3_) were purified according to the standard procedures described for each solvent.

*Synthesis of 2-amino-8-thia-1,5-di­aza­spiro­[4.5]dec-1-en-5-ium chloride hydrate* (**3**):

To a solution of 1.00 g (0.0053 mol) of β-(thio­morpholin-1-yl)propio­amidoxime (**1**) in 40 ml of CHCl_3_, 0.92 ml (0.0053 mol) of DIPEA were added. The reaction mixture was cooled to 272 K, and a solution of 1.01 g (0.00530 mol) of tosyl chloride in 4 ml of CHCl_3_ was added dropwise under stirring. The reaction mixture was stirred for 1 h at room temperature and was then heated and stirred at the reflux temperature of CHCl_3_ for 8 h until the completion of the reaction, the progress of the reaction being monitored by TLC. The formed white precipitate of the chloride hydrate **3** was filtered off and recrystallized from 2-PrOH solution. The yield of **3** was 1.01 g (84%), m.p. 575 K, *R*_f_ 0.08. Found, %: C 37.67, H 7.49. C_7_H_16_ClN_3_OS. Calculated, %: C 37.24, H 7.14. IR, cm^−1^: 1659 (ν C=N); 1612 [*d* C—N; *d* (H)2—O]; 670 (ν S—C); 3135, 3230, 3380, 3384 (ν H—O, ν H—N). ^1^H NMR, δ, ppm (*J*, Hz): 2.88 [*m*, 2H, S(CH_eq_)_2_], 3.14 [*m*, 2H, S(CH_ax_)_2_], 3.14 [*m*, 2H, N(+)CH_2_CH_2_]^§^, 3.37 (*br. s*, 2H, H_2_O), 3.62 [*m*, 2H, N(+)C(H_eq_)_2_], 3.74 [*m*, 2H, N(+)C(H*ax*)_2_], 3.88 [*t*, 2H, J = 7.0, N(+)CH_2_CH_2_], 7.48 *(br s*, 2H, NH_2_). The signals for the methyl­ene protons of the N(+)CH_2_CH_2_ group in **3** coincide with the signals of the S(CH_ax_)_2_ group at δ 3.14 ppm.

## Refinement

Crystal data, data collection and structure refinement details are summarized in Table 3 ▸. The positions of hydrogen atoms were calculated and included in the refinement in isotropic approximation using a riding model with *U*_iso_(H) = 1.5*U*_eq_(O) and 1.2*U*_eq_(*X*) for the other atoms.

## Supplementary Material

Crystal structure: contains datablock(s) 3_LT, 3_RT. DOI: 10.1107/S2056989022000111/hb8000sup1.cif

Structure factors: contains datablock(s) 3_LT. DOI: 10.1107/S2056989022000111/hb80003_LTsup2.hkl

Structure factors: contains datablock(s) 3_RT. DOI: 10.1107/S2056989022000111/hb80003_RTsup3.hkl

CCDC references: 2106799, 2106798

Additional supporting information:  crystallographic information; 3D view; checkCIF report

## Figures and Tables

**Table 1:** Hydrogen-bond geometry (Å, °) for the low-temperature structure[Chem scheme1]

*D*—H⋯*A*	*D*—H	H⋯*A*	*D*⋯*A*	*D*—H⋯*A*
N3—H3*A*⋯Cl1^i^	0.88	2.38	3.2560 (9)	175
N3—H3*B*⋯O1	0.88	1.95	2.7970 (11)	161
O1—H1*A*⋯Cl1	0.85	2.26	3.1042 (9)	175
O1—H1*B*⋯Cl1^ii^	0.85	2.27	3.1152 (9)	176
C5—H5*B*⋯N2^iii^	0.99	2.58	3.3778 (12)	138
C6—H6*A*⋯N2^iv^	0.99	2.55	3.3346 (12)	136

Symmetry codes: (i) x+{\script{1\over 2}}, -y+{\script{3\over 2}}, -z+1; (ii) -x, -y+1, -z+1; (iii) -x+1, y-{\script{1\over 2}}, -z+{\script{3\over 2}}; (iv) -x+{\script{3\over 2}}, y-{\script{1\over 2}}, z.

**Table 2:** Hydrogen-bond geometry (Å, °) for the room-temperature structure[Chem scheme1]

*D*—H⋯*A*	*D*—H	H⋯*A*	*D*⋯*A*	*D*—H⋯*A*
N3—H3*A*⋯Cl1^i^	0.86	2.42	3.2786 (18)	174
N3—H3*B*⋯O1^ii^	0.86	1.99	2.821 (2)	162
O1—H1*C*⋯Cl1	0.85	2.28	3.1244 (17)	173
O1—H1*D*⋯Cl1^iii^	0.85	2.29	3.1343 (17)	175
C6—H6*B*⋯N2^iv^	0.97	2.61	3.391 (2)	138

Symmetry codes: (i) x, y+1, z; (ii) x+{\script{1\over 2}}, -y+{\script{1\over 2}}, -z+1; (iii) -x+1, -y, -z+1; (iv) -x+{\script{1\over 2}}, y-{\script{1\over 2}}, z.

**Table 3:** Experimental details

	120 K	295 K
Crystal data		
Chemical formula	C_7_H_14_N_3_S^+^·Cl^−^·H_2_O	C_7_H_14_N_3_S^+^·Cl^−^·H_2_O
*M*_r_	225.74	225.74
Crystal system, space group	Orthorhombic, *P* *b* *c* *a*	Orthorhombic, *P* *b* *c* *a*
*a*, *b*, *c* (Å)	11.0360 (18), 10.1005 (16), 19.291 (3)	11.0924 (4), 10.1898 (4), 19.6434 (8)
*V* (Å^3^)	2150.4 (6)	2220.28 (15)
*Z*	8	8
Radiation type	Mo *K*α	Mo *K*α
μ (mm^−1^)	0.52	0.50
Crystal size (mm)	0.41 × 0.36 × 0.32	0.41 × 0.36 × 0.32

Data collection		
Diffractometer	Bruker APEXII CCD	Bruker D8 Quest PHOTON area detector
Absorption correction	Multi-scan (*SADABS*; Bruker, 2016 ▸)	Multi-scan (*SADABS*; Bruker, 2016 ▸)
*T*_min_, *T*_max_	0.633, 0.747	0.518, 0.746
No. of measured, independent and observed [*I* > 2σ(*I*)] reflections	29600, 5145, 3893	29622, 2969, 2269
*R*_int_	0.032	0.106
(sin θ/λ)_max_ (Å^−1^)	0.830	0.684

Refinement		
*R*[*F* ^2^ > 2σ(*F* ^2^)], *wR*(*F* ^2^), *S*	0.030, 0.087, 1.06	0.048, 0.124, 1.05
No. of reflections	5145	2969
No. of parameters	121	118
H-atom treatment	H-atom parameters constrained	H-atom parameters constrained
Δρ_max_, Δρ_min_ (e Å^−3^)	0.44, −0.34	0.29, −0.34

Computer programs: *APEX2* and *SAINT* (Bruker, 2016 ▸), *SHELXT* (Sheldrick, 2015*a* ▸), *SHELXL* (Sheldrick, 2015*b* ▸) and *OLEX2* (Dolomanov *et al.*, 2009 ▸).

## supplementary crystallographic information

### 2-Amino-8-thia-1,5-diazaspiro[4.5]dec-1-en-5-ium chloride monohydrate (3_LT) Crystal data

**Table:**

C_7_H_14_N_3_S^+^·Cl^−^·H_2_O	*D*_x_ = 1.395 Mg m^−^^3^
*M**_r_* = 225.74	Mo *K*α radiation, λ = 0.71073 Å
Orthorhombic, *P**b**c**a*	Cell parameters from 8488 reflections
*a* = 11.0360 (18) Å	θ = 2.8–35.6°
*b* = 10.1005 (16) Å	µ = 0.52 mm^−^^1^
*c* = 19.291 (3) Å	*T* = 120 K
*V* = 2150.4 (6) Å^3^	Prism, colourless
*Z* = 8	0.41 × 0.36 × 0.32 mm
*F*(000) = 960	

### 2-Amino-8-thia-1,5-diazaspiro[4.5]dec-1-en-5-ium chloride monohydrate (3_LT) Data collection

**Table:**

Bruker APEXII CCD diffractometer	3893 reflections with *I* > 2σ(*I*)
φ and ω scans	*R*_int_ = 0.032
Absorption correction: multi-scan (SADABS; Bruker, 2016)	θ_max_ = 36.2°, θ_min_ = 2.1°
*T*_min_ = 0.633, *T*_max_ = 0.747	*h* = −18→18
29600 measured reflections	*k* = −16→16
5145 independent reflections	*l* = −26→32

### 2-Amino-8-thia-1,5-diazaspiro[4.5]dec-1-en-5-ium chloride monohydrate (3_LT) Refinement

**Table:**

Refinement on *F*^2^	0 restraints
Least-squares matrix: full	Hydrogen site location: mixed
*R*[*F*^2^ > 2σ(*F*^2^)] = 0.030	H-atom parameters constrained
*wR*(*F*^2^) = 0.087	*w* = 1/[σ^2^(*F*_o_^2^) + (0.050*P*)^2^] where *P* = (*F*_o_^2^ + 2*F*_c_^2^)/3
*S* = 1.06	(Δ/σ)_max_ = 0.002
5145 reflections	Δρ_max_ = 0.44 e Å^−^^3^
121 parameters	Δρ_min_ = −0.34 e Å^−^^3^

### 2-Amino-8-thia-1,5-diazaspiro[4.5]dec-1-en-5-ium chloride monohydrate (3_LT) Special details

**Table:**

Geometry. All esds (except the esd in the dihedral angle between two l.s. planes) are estimated using the full covariance matrix. The cell esds are taken into account individually in the estimation of esds in distances, angles and torsion angles; correlations between esds in cell parameters are only used when they are defined by crystal symmetry. An approximate (isotropic) treatment of cell esds is used for estimating esds involving l.s. planes.

### 2-Amino-8-thia-1,5-diazaspiro[4.5]dec-1-en-5-ium chloride monohydrate (3_LT) Fractional atomic coordinates and isotropic or equivalent isotropic displacement parameters (Å^2^)

**Table:**

	*x*	*y*	*z*	*U*_iso_*/*U*_eq_	
Cl1	−0.00746 (2)	0.63396 (2)	0.39143 (2)	0.02000 (6)	
S1	0.70833 (2)	0.12361 (2)	0.74087 (2)	0.02033 (6)	
N1	0.55731 (6)	0.31961 (6)	0.64122 (3)	0.01183 (11)	
N2	0.53333 (6)	0.45906 (6)	0.62609 (4)	0.01348 (12)	
N3	0.38623 (7)	0.57594 (7)	0.56713 (4)	0.02087 (15)	
H3A	0.4200	0.6520	0.5782	0.025*	
H3B	0.3199	0.5747	0.5418	0.025*	
C1	0.43545 (7)	0.46295 (7)	0.58861 (4)	0.01473 (14)	
C2	0.38062 (8)	0.33120 (8)	0.57138 (5)	0.02009 (16)	
H2A	0.3594	0.3249	0.5216	0.024*	
H2B	0.3077	0.3134	0.5997	0.024*	
C3	0.48341 (7)	0.23868 (8)	0.58991 (4)	0.01713 (15)	
H3C	0.5320	0.2156	0.5485	0.021*	
H3D	0.4527	0.1564	0.6115	0.021*	
C4	0.51885 (7)	0.29562 (8)	0.71514 (4)	0.01528 (14)	
H4A	0.4304	0.3102	0.7189	0.018*	
H4B	0.5595	0.3611	0.7454	0.018*	
C5	0.54806 (8)	0.15791 (8)	0.74105 (4)	0.01851 (16)	
H5A	0.5063	0.0922	0.7114	0.022*	
H5B	0.5166	0.1479	0.7888	0.022*	
C6	0.73185 (8)	0.16153 (8)	0.65050 (4)	0.01875 (16)	
H6A	0.8190	0.1519	0.6393	0.022*	
H6B	0.6862	0.0975	0.6217	0.022*	
C7	0.69134 (7)	0.30023 (8)	0.63285 (4)	0.01590 (14)	
H7A	0.7345	0.3637	0.6632	0.019*	
H7B	0.7141	0.3200	0.5843	0.019*	
O1	0.16762 (6)	0.51532 (7)	0.50099 (3)	0.02415 (14)	
H1A	0.1157	0.5460	0.4727	0.036*	
H1B	0.1256	0.4764	0.5318	0.036*	

### 2-Amino-8-thia-1,5-diazaspiro[4.5]dec-1-en-5-ium chloride monohydrate (3_LT) Atomic displacement parameters (Å^2^)

**Table:**

	*U*^11^	*U*^22^	*U*^33^	*U*^12^	*U*^13^	*U*^23^
Cl1	0.02624 (11)	0.01477 (9)	0.01898 (10)	0.00288 (7)	0.00057 (7)	0.00344 (7)
S1	0.02337 (11)	0.01616 (10)	0.02145 (11)	0.00301 (7)	−0.00515 (8)	0.00290 (7)
N1	0.0134 (3)	0.0084 (2)	0.0137 (3)	−0.0004 (2)	−0.0002 (2)	−0.0008 (2)
N2	0.0158 (3)	0.0082 (3)	0.0165 (3)	0.0001 (2)	−0.0017 (2)	0.0006 (2)
N3	0.0217 (3)	0.0126 (3)	0.0283 (4)	0.0003 (3)	−0.0115 (3)	0.0011 (3)
C1	0.0149 (3)	0.0123 (3)	0.0171 (3)	−0.0004 (3)	−0.0010 (3)	−0.0010 (3)
C2	0.0198 (4)	0.0125 (3)	0.0279 (4)	−0.0015 (3)	−0.0091 (3)	−0.0019 (3)
C3	0.0215 (4)	0.0110 (3)	0.0189 (4)	−0.0006 (3)	−0.0058 (3)	−0.0036 (3)
C4	0.0183 (3)	0.0138 (3)	0.0137 (3)	0.0017 (3)	0.0035 (3)	0.0007 (3)
C5	0.0221 (4)	0.0153 (3)	0.0181 (4)	−0.0005 (3)	0.0025 (3)	0.0038 (3)
C6	0.0178 (4)	0.0169 (3)	0.0216 (4)	0.0057 (3)	0.0005 (3)	−0.0005 (3)
C7	0.0121 (3)	0.0163 (3)	0.0192 (4)	0.0010 (3)	0.0026 (3)	0.0024 (3)
O1	0.0161 (3)	0.0358 (4)	0.0205 (3)	−0.0016 (3)	−0.0015 (2)	0.0046 (3)

### 2-Amino-8-thia-1,5-diazaspiro[4.5]dec-1-en-5-ium chloride monohydrate (3_LT) Geometric parameters (Å, º)

**Table:**

S1—C5	1.8024 (9)	C3—H3C	0.9900
S1—C6	1.8038 (9)	C3—H3D	0.9900
N1—N2	1.4626 (9)	C4—H4A	0.9900
N1—C3	1.5210 (10)	C4—H4B	0.9900
N1—C4	1.5074 (10)	C4—C5	1.5128 (11)
N1—C7	1.5007 (10)	C5—H5A	0.9900
N2—C1	1.3003 (10)	C5—H5B	0.9900
N3—H3A	0.8800	C6—H6A	0.9900
N3—H3B	0.8800	C6—H6B	0.9900
N3—C1	1.3301 (10)	C6—C7	1.5093 (11)
C1—C2	1.4992 (11)	C7—H7A	0.9900
C2—H2A	0.9900	C7—H7B	0.9900
C2—H2B	0.9900	O1—H1A	0.8501
C2—C3	1.5125 (11)	O1—H1B	0.8499
			
C5—S1—C6	95.87 (4)	N1—C4—H4A	108.8
N2—N1—C3	106.89 (6)	N1—C4—H4B	108.8
N2—N1—C4	107.01 (5)	N1—C4—C5	113.60 (6)
N2—N1—C7	106.41 (6)	H4A—C4—H4B	107.7
C4—N1—C3	112.23 (6)	C5—C4—H4A	108.8
C7—N1—C3	112.85 (6)	C5—C4—H4B	108.8
C7—N1—C4	111.00 (6)	S1—C5—H5A	109.1
C1—N2—N1	106.89 (6)	S1—C5—H5B	109.1
H3A—N3—H3B	120.0	C4—C5—S1	112.66 (6)
C1—N3—H3A	120.0	C4—C5—H5A	109.1
C1—N3—H3B	120.0	C4—C5—H5B	109.1
N2—C1—N3	122.58 (7)	H5A—C5—H5B	107.8
N2—C1—C2	115.57 (7)	S1—C6—H6A	109.2
N3—C1—C2	121.84 (7)	S1—C6—H6B	109.2
C1—C2—H2A	111.5	H6A—C6—H6B	107.9
C1—C2—H2B	111.5	C7—C6—S1	111.87 (6)
C1—C2—C3	101.15 (6)	C7—C6—H6A	109.2
H2A—C2—H2B	109.4	C7—C6—H6B	109.2
C3—C2—H2A	111.5	N1—C7—C6	112.88 (6)
C3—C2—H2B	111.5	N1—C7—H7A	109.0
N1—C3—H3C	111.2	N1—C7—H7B	109.0
N1—C3—H3D	111.2	C6—C7—H7A	109.0
C2—C3—N1	102.94 (6)	C6—C7—H7B	109.0
C2—C3—H3C	111.2	H7A—C7—H7B	107.8
C2—C3—H3D	111.2	H1A—O1—H1B	104.5
H3C—C3—H3D	109.1		
			
S1—C6—C7—N1	−64.89 (8)	C3—N1—C4—C5	68.11 (8)
N1—N2—C1—N3	178.26 (7)	C3—N1—C7—C6	−66.08 (9)
N1—N2—C1—C2	−1.35 (10)	C4—N1—N2—C1	−103.39 (7)
N1—C4—C5—S1	61.65 (8)	C4—N1—C3—C2	91.71 (8)
N2—N1—C3—C2	−25.32 (8)	C4—N1—C7—C6	60.89 (8)
N2—N1—C4—C5	−174.93 (6)	C5—S1—C6—C7	56.71 (7)
N2—N1—C7—C6	176.99 (6)	C6—S1—C5—C4	−55.24 (7)
N2—C1—C2—C3	−14.59 (10)	C7—N1—N2—C1	137.87 (7)
N3—C1—C2—C3	165.80 (8)	C7—N1—C3—C2	−141.97 (7)
C1—C2—C3—N1	22.86 (8)	C7—N1—C4—C5	−59.21 (8)
C3—N1—N2—C1	17.04 (8)		

### 2-Amino-8-thia-1,5-diazaspiro[4.5]dec-1-en-5-ium chloride monohydrate (3_LT) Hydrogen-bond geometry (Å, º)

**Table:**

*D*—H···*A*	*D*—H	H···*A*	*D*···*A*	*D*—H···*A*
N3—H3*A*···Cl1^i^	0.88	2.38	3.2560 (9)	175
N3—H3*B*···O1	0.88	1.95	2.7970 (11)	161
O1—H1*A*···Cl1	0.85	2.26	3.1042 (9)	175
O1—H1*B*···Cl1^ii^	0.85	2.27	3.1152 (9)	176
C5—H5*B*···N2^iii^	0.99	2.58	3.3778 (12)	138
C6—H6*A*···N2^iv^	0.99	2.55	3.3346 (12)	136

Symmetry codes: (i) *x*+1/2, −*y*+3/2, −*z*+1; (ii) −*x*, −*y*+1, −*z*+1; (iii) −*x*+1, *y*−1/2, −*z*+3/2; (iv) −*x*+3/2, *y*−1/2, *z*.

### 2-Amino-8-thia-1,5-diazaspiro[4.5]dec-1-en-5-ium chloride monohydrate (3_RT) Crystal data

**Table:**

C_7_H_14_N_3_S^+^·Cl^−^·H_2_O	*D*_x_ = 1.351 Mg m^−^^3^
*M**_r_* = 225.74	Mo *K*α radiation, λ = 0.71073 Å
Orthorhombic, *P**b**c**a*	Cell parameters from 8223 reflections
*a* = 11.0924 (4) Å	θ = 2.8–28.8°
*b* = 10.1898 (4) Å	µ = 0.50 mm^−^^1^
*c* = 19.6434 (8) Å	*T* = 295 K
*V* = 2220.28 (15) Å^3^	Prism, colourless
*Z* = 8	0.41 × 0.36 × 0.32 mm
*F*(000) = 960	

### 2-Amino-8-thia-1,5-diazaspiro[4.5]dec-1-en-5-ium chloride monohydrate (3_RT) Data collection

**Table:**

Bruker D8 Quest PHOTON area detector diffractometer	2269 reflections with *I* > 2σ(*I*)
Graphite monochromator	*R*_int_ = 0.106
phi and ω scans	θ_max_ = 29.1°, θ_min_ = 2.1°
Absorption correction: multi-scan (SADABS; Bruker, 2016)	*h* = −15→14
*T*_min_ = 0.518, *T*_max_ = 0.746	*k* = −11→13
29622 measured reflections	*l* = −26→26
2969 independent reflections	

### 2-Amino-8-thia-1,5-diazaspiro[4.5]dec-1-en-5-ium chloride monohydrate (3_RT) Refinement

**Table:**

Refinement on *F*^2^	Primary atom site location: dual
Least-squares matrix: full	Hydrogen site location: mixed
*R*[*F*^2^ > 2σ(*F*^2^)] = 0.048	H-atom parameters constrained
*wR*(*F*^2^) = 0.124	*w* = 1/[σ^2^(*F*_o_^2^) + (0.0532*P*)^2^ + 0.7125*P*] where *P* = (*F*_o_^2^ + 2*F*_c_^2^)/3
*S* = 1.05	(Δ/σ)_max_ = 0.001
2969 reflections	Δρ_max_ = 0.29 e Å^−^^3^
118 parameters	Δρ_min_ = −0.34 e Å^−^^3^
0 restraints	

### 2-Amino-8-thia-1,5-diazaspiro[4.5]dec-1-en-5-ium chloride monohydrate (3_RT) Special details

**Table:**

Geometry. All esds (except the esd in the dihedral angle between two l.s. planes) are estimated using the full covariance matrix. The cell esds are taken into account individually in the estimation of esds in distances, angles and torsion angles; correlations between esds in cell parameters are only used when they are defined by crystal symmetry. An approximate (isotropic) treatment of cell esds is used for estimating esds involving l.s. planes.

### 2-Amino-8-thia-1,5-diazaspiro[4.5]dec-1-en-5-ium chloride monohydrate (3_RT) Fractional atomic coordinates and isotropic or equivalent isotropic displacement parameters (Å^2^)

**Table:**

	*x*	*y*	*z*	*U*_iso_*/*U*_eq_	
Cl1	0.50712 (5)	−0.13596 (5)	0.60611 (3)	0.04965 (17)	
S1	0.28950 (5)	0.12409 (5)	0.73957 (3)	0.04811 (17)	
N1	0.44413 (12)	0.31778 (13)	0.64306 (7)	0.0269 (3)	
N2	0.46759 (13)	0.45621 (14)	0.62725 (8)	0.0308 (3)	
N3	0.61220 (16)	0.57229 (17)	0.56826 (10)	0.0481 (5)	
H3A	0.5789	0.6458	0.5787	0.058*	
H3B	0.6764	0.5712	0.5437	0.058*	
C1	0.52107 (19)	0.23660 (19)	0.59441 (11)	0.0433 (5)	
H1A	0.5539	0.1599	0.6170	0.052*	
H1B	0.4746	0.2086	0.5552	0.052*	
C2	0.61951 (18)	0.32998 (19)	0.57400 (12)	0.0463 (5)	
H2A	0.6923	0.3153	0.6003	0.056*	
H2B	0.6382	0.3225	0.5259	0.056*	
C3	0.56420 (16)	0.45980 (17)	0.59016 (9)	0.0325 (4)	
C4	0.47724 (18)	0.29721 (19)	0.71662 (9)	0.0364 (4)	
H4A	0.4345	0.3610	0.7442	0.044*	
H4B	0.5629	0.3132	0.7222	0.044*	
C5	0.4484 (2)	0.1611 (2)	0.74256 (11)	0.0438 (5)	
H5A	0.4764	0.1533	0.7892	0.053*	
H5B	0.4918	0.0972	0.7154	0.053*	
C6	0.27154 (18)	0.1587 (2)	0.65017 (11)	0.0442 (5)	
H6A	0.3183	0.0961	0.6240	0.053*	
H6B	0.1875	0.1479	0.6378	0.053*	
C7	0.31144 (16)	0.2961 (2)	0.63200 (10)	0.0384 (4)	
H7A	0.2924	0.3128	0.5846	0.046*	
H7B	0.2666	0.3585	0.6594	0.046*	
O1	0.33147 (14)	−0.01611 (18)	0.49843 (8)	0.0577 (5)	
H1C	0.3755	−0.0450	0.5305	0.087*	
H1D	0.3711	0.0261	0.4686	0.087*	

### 2-Amino-8-thia-1,5-diazaspiro[4.5]dec-1-en-5-ium chloride monohydrate (3_RT) Atomic displacement parameters (Å^2^)

**Table:**

	*U*^11^	*U*^22^	*U*^33^	*U*^12^	*U*^13^	*U*^23^
Cl1	0.0635 (4)	0.0357 (3)	0.0498 (3)	0.0080 (2)	0.0013 (2)	0.0113 (2)
S1	0.0550 (3)	0.0369 (3)	0.0524 (3)	−0.0082 (2)	0.0144 (2)	0.0064 (2)
N1	0.0311 (7)	0.0193 (6)	0.0302 (7)	0.0009 (5)	0.0013 (5)	−0.0024 (5)
N2	0.0360 (8)	0.0194 (7)	0.0370 (8)	0.0012 (6)	0.0059 (6)	0.0009 (6)
N3	0.0517 (10)	0.0287 (8)	0.0640 (12)	−0.0021 (7)	0.0241 (8)	0.0024 (8)
C1	0.0537 (12)	0.0260 (9)	0.0500 (11)	0.0012 (8)	0.0201 (9)	−0.0096 (8)
C2	0.0443 (11)	0.0301 (10)	0.0644 (13)	0.0043 (8)	0.0196 (9)	−0.0042 (9)
C3	0.0358 (9)	0.0261 (8)	0.0355 (9)	0.0015 (7)	0.0037 (7)	−0.0016 (7)
C4	0.0446 (10)	0.0318 (9)	0.0326 (9)	−0.0055 (7)	−0.0092 (7)	0.0009 (7)
C5	0.0549 (12)	0.0363 (10)	0.0401 (10)	−0.0013 (9)	−0.0076 (9)	0.0111 (8)
C6	0.0400 (11)	0.0384 (11)	0.0540 (12)	−0.0131 (8)	−0.0035 (9)	−0.0026 (9)
C7	0.0319 (9)	0.0390 (10)	0.0444 (10)	−0.0051 (8)	−0.0081 (7)	0.0061 (8)
O1	0.0387 (8)	0.0872 (13)	0.0473 (8)	−0.0039 (8)	−0.0054 (6)	0.0084 (8)

### 2-Amino-8-thia-1,5-diazaspiro[4.5]dec-1-en-5-ium chloride monohydrate (3_RT) Geometric parameters (Å, º)

**Table:**

S1—C5	1.804 (2)	C2—H2B	0.9700
S1—C6	1.802 (2)	C2—C3	1.492 (2)
N1—N2	1.4676 (19)	C4—H4A	0.9700
N1—C1	1.525 (2)	C4—H4B	0.9700
N1—C4	1.506 (2)	C4—C5	1.512 (3)
N1—C7	1.504 (2)	C5—H5A	0.9700
N2—C3	1.296 (2)	C5—H5B	0.9700
N3—H3A	0.8600	C6—H6A	0.9700
N3—H3B	0.8600	C6—H6B	0.9700
N3—C3	1.335 (2)	C6—C7	1.511 (3)
C1—H1A	0.9700	C7—H7A	0.9700
C1—H1B	0.9700	C7—H7B	0.9700
C1—C2	1.503 (3)	O1—H1C	0.8500
C2—H2A	0.9700	O1—H1D	0.8499
			
C6—S1—C5	95.67 (9)	N1—C4—H4A	108.9
N2—N1—C1	106.83 (13)	N1—C4—H4B	108.9
N2—N1—C4	107.07 (13)	N1—C4—C5	113.56 (15)
N2—N1—C7	106.50 (13)	H4A—C4—H4B	107.7
C4—N1—C1	112.92 (15)	C5—C4—H4A	108.9
C7—N1—C1	112.18 (14)	C5—C4—H4B	108.9
C7—N1—C4	110.91 (14)	S1—C5—H5A	109.0
C3—N2—N1	107.02 (13)	S1—C5—H5B	109.0
H3A—N3—H3B	120.0	C4—C5—S1	112.80 (14)
C3—N3—H3A	120.0	C4—C5—H5A	109.0
C3—N3—H3B	120.0	C4—C5—H5B	109.0
N1—C1—H1A	111.1	H5A—C5—H5B	107.8
N1—C1—H1B	111.1	S1—C6—H6A	109.1
H1A—C1—H1B	109.1	S1—C6—H6B	109.1
C2—C1—N1	103.32 (15)	H6A—C6—H6B	107.9
C2—C1—H1A	111.1	C7—C6—S1	112.28 (14)
C2—C1—H1B	111.1	C7—C6—H6A	109.1
C1—C2—H2A	111.4	C7—C6—H6B	109.1
C1—C2—H2B	111.4	N1—C7—C6	112.86 (15)
H2A—C2—H2B	109.3	N1—C7—H7A	109.0
C3—C2—C1	101.88 (15)	N1—C7—H7B	109.0
C3—C2—H2A	111.4	C6—C7—H7A	109.0
C3—C2—H2B	111.4	C6—C7—H7B	109.0
N2—C3—N3	122.34 (16)	H7A—C7—H7B	107.8
N2—C3—C2	115.74 (16)	H1C—O1—H1D	112.9
N3—C3—C2	121.91 (16)		

### 2-Amino-8-thia-1,5-diazaspiro[4.5]dec-1-en-5-ium chloride monohydrate (3_RT) Hydrogen-bond geometry (Å, º)

**Table:**

*D*—H···*A*	*D*—H	H···*A*	*D*···*A*	*D*—H···*A*
N3—H3*A*···Cl1^i^	0.86	2.42	3.2786 (18)	174
N3—H3*B*···O1^ii^	0.86	1.99	2.821 (2)	162
O1—H1*C*···Cl1	0.85	2.28	3.1244 (17)	173
O1—H1*D*···Cl1^iii^	0.85	2.29	3.1343 (17)	175
C6—H6*B*···N2^iv^	0.97	2.61	3.391 (2)	138

Symmetry codes: (i) *x*, *y*+1, *z*; (ii) *x*+1/2, −*y*+1/2, −*z*+1; (iii) −*x*+1, −*y*, −*z*+1; (iv) −*x*+1/2, *y*−1/2, *z*.
